# Understanding the physical processes behind DNA-DNA proximity ligation assays

**DOI:** 10.21203/rs.3.rs-7859870/v1

**Published:** 2025-12-02

**Authors:** Bernardo J. Zubillaga Herrera, Amit Das, Linden Burack, Ailung Wang, Michele Di Pierro

**Affiliations:** a Center for Theoretical Biological Physics, Northeastern University, Boston, Massachusetts 02115, United States.; b Department of Physics, Northeastern University, Boston, Massachusetts 02115, United States.; c Department of Biochemical Engineering and Biotechnology, Indian Institute of Technology Delhi, Hauz Khaz, New Delhi 100 016, India.; d ByteDance Ltd., New York, New York 10036, United States.

**Keywords:** Chromosome Conformation Capture, Hi-C, Proximity Ligation Assays, Contact Maps, DNA, DNA Structure and Organization

## Abstract

In the last decade, DNA-DNA proximity ligation assays opened powerful new ways to study the 3D organization of genomes and have become a mainstay experimental technology. Yet many aspects of these experiments remain poorly understood. We study the inner workings of DNA-DNA proximity ligation assays through numerical experiments and theoretical modeling. Chromosomes are modeled at nucleosome resolution and evolved in time via molecular dynamics. A virtual Hi-C experiment reproduces, in-silico, the different steps of the Hi-C protocol, including: crosslinking of chromatin to an underlying proteic matrix, enzymatic digestion of DNA, and subsequent proximity ligation of DNA open ends. The protocol is simulated on ensembles of different structures as well as individual structures, enabling the construction of ligation maps and the calculation of ligation probabilities as functions of genomic and Euclidean distance. The methods help to assess the effect of the many variables of the Hi-C experiment and of subsequent data processing methods on the quality of the final results.

## 1. INTRODUCTION

Experimental studies on 3D chromatin organization reveal the complexity of large-scale, spatial arrangements of DNA within the nucleus. This intricate organization is thought to be linked to key genomic processes like the machineries of transcription and regulation, DNA replication, repair, and overall genome function, often mediated by mechanisms such as loop extrusion or enhancer-promoter contacts. Chromosome conformation capture (3C) and related techniques enable the study of chromatin architecture by quantifying contact frequencies between genomic loci that are in close physical proximity within the 3D nuclear space, even if they separated by large genomic distances.^1–10^

Hi-C, a successful 3C technology, quantifies interactions between all possible pairs of genomic loci at a given resolution. Its strength lies in the comprehensive detection of chromatin contacts through proximity ligation combined with high-throughput paired-end sequencing. The resulting ligation maps reveal a wealth of complexity through checkerboard patterns, and structural features such as domains, loops, and compartments. By processing billions of paired-end reads, Hi-C enables a detailed and global view of chromosome architecture^1–7,11–27^.

Ensemble Hi-C measures the 3D contact frequency between pairs of genomic fragments by averaging over populations of cells, producing ligation maps that reflect aggregate proximity contacts. In contrast, single-cell Hi-C (scHi-C) captures chromatin interactions at the level of individual cells, revealing that chromosomes in single cells can exhibit domain-like structures, particularly at the Megabase scale.^3,28–31^ Micro-C furthers fine-scale resolution by probing chromatin structure down to single nucleosome resolution.^32–34^

Comparative in situ Hi-C studies across eukaryotic species have uncovered two major chromosomal-scale genome architectures.^35^ Hi-C has also enabled studies of chromatin organization reprogramming during early development stages after fertilization.^36^ Recently, PaleoHi-C revealed remarkable preservation of 3D genome architecture in ancient samples, successfully reconstructing chromatin interactions from a 52,000-year-old woolly mammoth (*Mammuthus primigenius*), establishing the applicability of Hi-C on extinct species.^37^

In Hi-C experiments, chromatin is first crosslinked with an agent like formaldehyde to preserve spatial proximity between genomic regions. Crosslinked DNA is then fragmented through enzymatic digestion. Fragment ends are labeled with biotin, and those that are in close physical proximity may undergo ligation. After purification and shearing of DNA, the ligated pair-end products are then sequenced. The sequenced pair-ends collectively reveal chromatin interactions and the contacts populate the experiment’s output: the ligation map.

Despite successfully revealing genome architecture, several aspects of Hi-C experiments remain poorly understood.^38–40^ Theoretical modeling, simulations and experiments have addressed some of these questions. For example, the effect of polymer collapse on contact probabilities due to irreversible crosslinking between monomers for varying crosslinker concentrations were numerically modelled, Correspondingly, experimental contact probability curves were measured over time under different crosslinking conditions to study the evolution of polymer concentration during crosslinking-induced collapse.^41^ Also, theoretical models, based on chromatin polymer models, were developed to better understand the capability and limitations of experimental methods such as Hi-C.^42^

**Figure 1.**
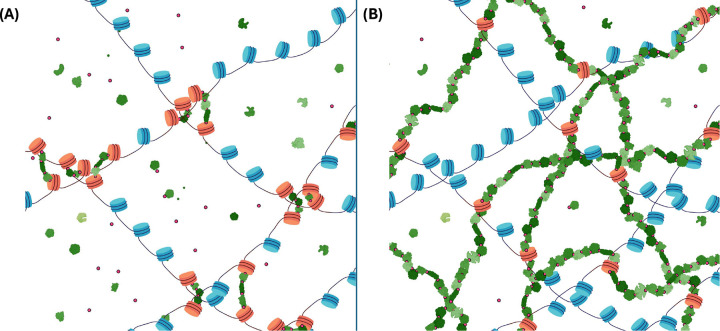
Two limiting cases of DNA crosslinking: Schematic representation of two conceptual extremes of crosslinking, with DNA depicted as chains of nucleosomes (blue), crosslinking agents such as formaldehyde as small red dots and proteins varying shades of green. Crosslinking occurs between the histones and proteins, as well among proteins themselves. Crosslinked nucleosomes are shown in red. ***(A) Short-range protein bridges:*** DNA is crosslinked via short-range protein bridges. This configuration permits a certain freedom of motion of the crosslinked nucleosomes within chromatin. Bridges shown include up to 3 proteins. ***(B) Nuclear protein matrix:*** DNA crosslinks to the scaffold provided by the percolated protein polymer network that spans physical extent of the nucleus, analogous to the cytoskeleton. The protein meshwork represents the long-range limit of protein-bridging, enabling higher-order chromatin interaction and providing structural integrity and rigidity to the nucleus. Created in BioRender. Zubillaga, B. (2025) https://BioRender.com/aixb5aa

An interesting outstanding question concerns crosslinking, owing to different chemical efficiencies for direct crosslinking between DNA-DNA, DNA-protein, or protein-protein interactions.^39^ One possibility suggests that spatially adjacent chromatin segments are covalently bonded via short-range protein bridges. If this is the case, the precise length scale of such bridges remains unclear. An alternative hypothesis suggests that DNA crosslinks to an underlying protein matrix that percolates across the spatial extent of the nucleus, with long-range protein bridges mediating higher-order DNA-DNA interactions.^39,43–45^ Supporting this idea, some studies report evidence for a nuclear matrix organized as a polymer meshwork analogous to the cytoskeleton, although its function and existence remain an unsettled matter.^43–51^ Fig. 1 illustrates two limiting cases: crosslinking via short-range protein bridges (Fig. 1(A)), and the nuclear protein matrix (Fig. 1(B)).

Open questions about the experiment’s chemical kinetics reveal uncertainty about effects of crosslinking agents, enzymatic digestion, and proximity ligation on Hi-C maps. Variations in the effective rates of these reactions, combined with the non-equilibrium diffusive motion of digested chromatin fragments, as well as differences in chromatin accessibility and local density, may all influence the final Hi-C maps. This demands careful inquiry for the sake of the accurate interpretation of experimental results.

Ligation frequency in Hi-C follows characteristic power-law decay with respect to genomic distance, reflecting complex long-range interactions in chromatin. This behavior is illustrated by the map’s checkerboard patterns. However, a relevant question remains: how does ligation probability depend on the 3D Euclidean distance between fragment ends? Specifically, what is the probability that two fragment ends, initially separated by a given Euclidean distance prior to digestion, will ligate as a function of said distance? This unexplored dependance would help bridge the conceptual gap between physical chromatin conformations and Hi-C-derived contact data.

Maps are usually subjected to numerical post-processing using ad-hoc computational algorithms.^38^ Among these, matrix balancing methods -such as Knight-Ruiz (KR) normalization-are widely used to treat Hi-C maps with the purported intent of correcting systematic biases in the data.^52^ However, the effect of these normalization methods on raw data and our interpretation of post-processed maps demands warrant closer scrutiny.

To address some of these questions, we probe the inner workings of DNA-DNA proximity ligation assays through numerical experiments. We define an *in-silico* protocol that emulates Hi-C experiments by modeling the sequence of chemical reactions involved. This virtual Hi-C framework mimics crosslinking of DNA to a percolating protein matrix, where DNA-DNA interactions are mediated by long-range protein bridges. The protocol then effectively models DNA digestion by restriction enzymes or endonucleases, followed by and proximity ligation of free fragment ends.

Chromosome structures at nucleosome resolution are evolved in time with molecular dynamics. Ligation events recorded throughout the simulation then populate ligation maps, the output of the in-silico experiment. These synthetic maps showcase features such as plaid patterns, domains and compartments, in agreement with experimental Hi-C maps.

In-silico Hi-C offers control over key chemical kinetic parameters -such as crosslinking, digestion, and ligation efficiencies- providing a window for the systematic investigation of their effects on ligation maps. The protocol can probe proximity ligation assays over ensembles of chromatin structures (ensemble Hi-C) and single structures (scHi-C). It allows repetitions of in-silico experiments on the same underlying structure, a capability not feasible in real scHi-C experiments given the irreversible destruction of DNA.

In addition to probing these chemical-kinetic, non-equilibrium effects on Hi-C maps, we address the outstanding question of how ligation probability depends on initial 3D Euclidean distance between loci pairs in their native conformations. Furthermore, we explore effects of numerical algorithms -specifically Knight-Ruiz matrix balancing- on Hi-C maps generated from in-silico experiments. This analysis enables a critical assessment of the influence of data postprocessing on the final interpretation of 3C data.

## 2. IN-SILICO PROTOCOL FOR PROXIMITY LIGATION ASSAYS

We model ensembles of structures 1.1 Mb in length, representative of chromosome 7 in human lymphoblastoid cells (genomic region: 95.4 to 96.5 Mbp). Each structure is modeled at 200 bp resolution, yielding a total of 5500 beads per conformation.^53^ Each bead represents a nucleosome, comprising ~150 bp of wrapped DNA plus ~50 bp of linker DNA. Prior to in-silico simulation of Hi-C, all structures undergo a preprocessing step in which the FIRE (Fast Inertial Relaxation Engine) algorithm is used to relax them into energetically optimized states.^54^ These energy-minimized conformations form the starting ensemble for the in-silico Hi-C simulations.

**Figure 2.**
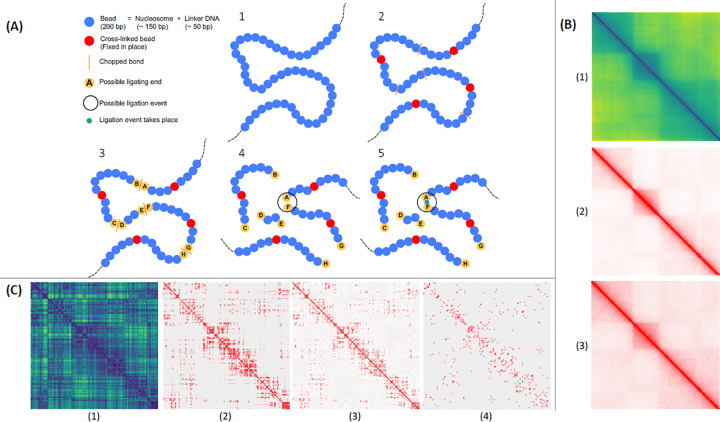
In-silico protocol at nucleosome resolution. ***(A) Basic steps:*** 1) Initial, native structure at nucleosome resolution (200bp). 2) Nucleosomes crosslink to protein matrix, fixed in place (in red). Bonds are enzymatically digested (cuts in orange). 3) Digested fragment ends (in yellow, labeled A-H) are free to ligate. 4) Structure evolves in time with molecular dynamics. 5) Ends A and F come into physical proximity (circled). 6) Ends A and F ligate (green) with a given probability rate. ***(B) Ensemble-based map:*** Maps are computed over an ensemble of 5,000 different structures modeling a 1.1Mbp region of human chromosome 7 (95.4 to 96.5 Mbp) at 200bp resolution. (1) Average distance map (in [nm]), averaged over native ensemble. (2) Average contact map from the native ensemble. Contacts are defined as beads pairs within threshold distance of *r* = 1.5*σ*, with *σ* = 10 [*nm*] (the Lennard-Jones length-scale parameter). (3) Average ligation map from simulations with 500 digested bonds, 500 crosslinked nucleosomes per structure, and a ligation rate *p* = 10^−2^ *p*_*o*_, where *p*_*o*_ = 1/*τ*, and *τ* = 2.2678 ± 0.0008 [*μs*]. Ligation is permitted when two fragment ends are within *r* = 1.5*σ*. Correspondence between average distance and contact maps with the ligation map shows the successful reproduction of structural features of the native ensemble, including checkerboard patterns, domains and compartments. ***(C) Single-structure maps (scHi-C):*** Maps derived from in-silico Hi-C on single native stucture. (1) Distance map of initial structure (in [nm]). (2) Contact map of initial structure based on a broader threshold *r* = 7.5*σ* for enhanced structural comparison. (3) Average ligation map from 5,000 scHi-C repetitions on the same initial structure, using the same of digestion, crosslinking and ligation parameters as in (B), and a threshold *r* = 1.5*σ*. (4) Ligation map from a single in-silico scHi-C iteration, shown at 10Kbp resolution for visual clarity, given the map’s sparsity at 200bp resolution (contrasted with Fig. 2.(C).(3)). Structural correspondence between distance, contact maps and ligation maps is apparent. The scHi-C protocol reproduces features of native structure and intimations of domains. It enables repeated measurements on the same structure, aggregating over sparse single-iteration map, a capability that is impossible in experimental scHi-C, where single cells are single-use.

Figure 2 illustrates the basic steps of the in-silico Hi-C for an individual sample. In Fig. 2.(A).(1), a native conformation is shown at nucleosome resolution, prior to crosslinking, digestion and ligation. In Fig. 2.(A).(2), a subset of nucleosomes (shown in red), are randomly selected and crosslinked to a nuclear protein matrix, according to a specified crosslinking efficiency. A fraction of bonds are cleaved, picked at random according to an enzymatic digestion efficiency, are cleaved; these cuts are depicted by straight orange lines. The protein matrix’s network-like scaffold confers a measure of structural rigidity to the nucleus. Within the in-silico framework, nucleosomes crosslinked to the matrix are treated as immobilized – that is, their motions are completely arrested and their positions fixed throughout the simulation.

Following crosslinking and digestion, the resulting chromatin segments possess free ends (labeled A through H and shown in yellow in Fig. 2.(A).(3)), which can undergo ligation under physical proximity. The crosslinked and digested structure is evolved over time with Langevin dynamics, with motion governed by a Hamiltonian incorporating Lennard-Jones interactions, finitely extensible nonlinear elastic (FENE) bonds and angular potentials.

During the simulation, if any pair of fragment ends comes within a threshold spatial distance, a ligation event may occur according to a ligation rate (probability of ligation per unit time). Successful ligation events are recorded as contacts and populate the ligation map.

In-silico modeling of enzymatic digestion as a stochastic process -where bonds are selected uniformly at random and cleaved to produce a randomly fragmented polymer- is supported by statistical analysis of human genomic sequences (see Supplementary Information, Figs. S1 and S2). We analyzed the T2T reference genome for restriction sites corresponding to 4-cutter enzyme MboI and to 6-cutter enzymes HindIII and NcoI. We found that the sites are uniformly distributed along the genome, and that distances between successive restriction sites follow an exponential distribution. These findings are consistent with uniform, memoryless digestion process in accordance with our random cleavage model.

This in-silico protocol enables calculations of ligation maps and ligation frequencies as functions of both genomic and Euclidean distances. It provides a framework for the systematic characterization of the underlying chemical kinetics of the Hi-C experiment, namely the effects of crosslinking, enzymatic digestion, and ligation efficiencies on the resulting maps. It allows for a controlled assessment of numerical post-processing algorithms (e.g., Knight-Ruiz matrix balancing) on ligation maps against raw, unprocessed data, offering insights on how numerical methods influence data interpretation.

## 3. RESULTS

### Crosslinking to a percolating protein matrix successfully recapitulates ensemble and single-cell Hi-C features.

Fig. 2 presents results from the in-silico Hi-C protocol applied to the ensemble of native structures at a nucleosome resolution. The average distance map of the ensemble (Fig. 2.(B).(1)) captures key structural features observed in experimental Hi-C maps, including: enriched interactions along the diagonal -due to frequent local interactions between nucleosomes at short genomic distances- as well as checkerboard patterns, domains and compartments. These features are also present in the ensemble-averaged contact map (Fig. 2.(B).(2)), where a contact is defined as a pair of loci separated by a Euclidean distance below a specified threshold. The final ligation map (Fig. 2.(B).(3)), produced after simulating crosslinking, digestion and proximity ligation across the ensemble, closely resembles the structure of both the average distance and contact maps. This strong correspondence demonstrates that in-silico Hi-C with crosslinks to a percolating proteic nuclear scaffold can faithfully reproduce the spatial organization typical of ensemble Hi-C experiments.

In-silico Hi-C can also simulate scHi-C methods, enacting the protocol on a single native conformation, as shown in Fig. 2.(C). An interesting possibility of this numerical approach, in contrast to scHi-C experiments, is the ability to repeat the protocol multiple times over the same initial structure. This allows for the aggregation of results to produce richer ligation maps with higher contact counts. This, of course, is a luxury not afforded by real scHi-C experiments, where each cell can only be measured once, severely limiting the contact yield of the final maps. Fig. 2.(C).(1) shows the distance map of a single structure, revealing intimations of domains and compartments. These features are reflected in the corresponding contact map (Fig. 2.(C).(2)), although, as expected for a single structure, the contact map is considerably sparse, especially when compared to its ensemble-averaged counterpart in Fig. 2.(B).(2).

Fig. 2.(C).(3) shows the ligation map resulting by repeating the in-silico protocol multiple times over the same initial structure and aggregating the results. In each repetition, the selection of crosslinked loci and digested bonds, as well as the subsequent physical evolution and ligation events, are sampled independently. This repetition enables richer sampling of possible outcomes while preserving the underlying structure, leading to an effective increase of contact density. In contrast, Figure 2(C)(4) shows the ligation map from a single iteration of the protocol on the same structure, which is much sparser and less informative. This clearly shows that by aggregating repeated experiments on the same conformation, structural features such as intimations of domains become more apparent—an advantage not afforded by real scHi-C, where each cell can only be probed once. The comparison between ensemble Hi-C and scHi-C maps in Figs. 2(B) and 2(C), respectively, shows a key difference observed in experimental data: ensemble maps display higher contact density and smoother features, while scHi-C maps are systematically sparser due to cell-to-cell variability in chromosome conformation.

**Figure 3.**
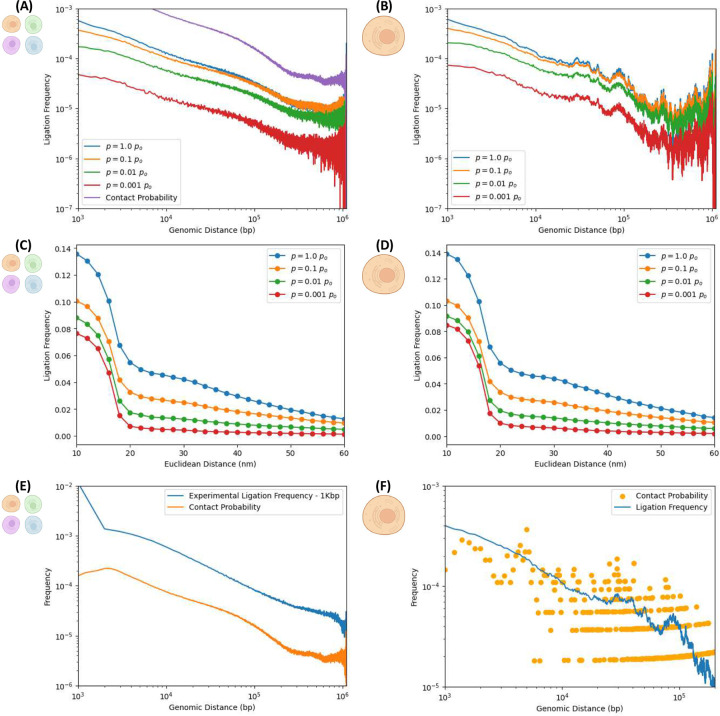
Dependence of ligation frequencies on genomic and Euclidean distances. Left and right columns correspond to numerical experiments simulating ensemble and single-cell Hi-C, resp***. (A and B) Ligation frequency as a function of genomic distance for different ligation rates*.** Ligation frequencies exhibit characteristic power-law scaling (typical of experimental maps) for different ligation rates “*p*”, capturing long-range structural features as domains and compartments. Results shown for different ligation rates span a few orders of magnitude both for: (A) ensemble Hi-C calculations (on 5000 different structures) and (B) scHi-C calculations (aggregating 5000 realizations on a single structure). For comparison, the average contact probability of the ensemble is shown.^53^ In (A), ligation frequencies closely resemble the contact probability of the native ensemble of initial structures. ***(C and D) Ligation frequency as a function of the 3D Euclidean distance between nucleosomes pairs for different ligation rates:*** Each data point reflects the frequency with which a pair of free fragment ends, initially separated by a Euclidean distance *d* in the native structure, comes within proximity (*r* = 1.5*σ* = 15 nm) and ligates during time evolution. The resulting sigmoidal curves suggest the presence of a characteristic length scale -on the order of ~20 [nm]- within which ligation events predominantly occur. *(C)* Ensemble Hi-C (5000 structures); *(D)* scHi-C (5000 iterations over the same structure). These curves are not probability density functions and are not subject to normalization. ***(E) Agreement with experimental contact probability:*** The ensemble contact probability over 5000 native structures representative of a 1.1Mbp region of chromosome 7 agrees with corresponding experimental results over same region at 1Kbp resolution, sharing similar exponents. ***(F) Averaging effect in scHi-C simulations:*** Average ligation frequency from 5000 iterations of scHi-C numerical experiment on the same initial structure is contrasted with the structure’s underlying contact probability. Averaging across iterations smooths out noise, due to statistical independence in bond selection, crosslinking, and stochastic dynamics. Created in BioRender. Zubillaga, B. (2025) https://BioRender.com/6afacus

Further evidence of consistency with experiment is provided by the scaling of ligation probability as a function of genomic separation between pairs of loci, shown in Fig. 3.(A). It is well-established that contact probabilities in Hi-C experiments follow characteristic power-law decays, reflecting the hierarchical organization of chromatin complexity into domains, loops and compartments. Fig. 3.(A) shows the ligation frequency versus genomic distance for the native ensemble, whereas Fig. 3.(B) does so for a single structure sampled many times (as in Fig. 2.(C)). In both cases, curves are ordered according to the ligation rate, which is varied across orders of magnitude. The in-silico protocol reproduces the typical scaling behavior of the ligation frequency observed in experiments. Notably, the ensemble-based ligation frequency curves in Fig. 3.(A) track the expected scaling associated with the native conformation’s contact maps. Finally, Fig. 3.(E) shows excellent agreement between in-silico results and actual experimental Hi-C ligation frequencies measures at a 1Kbp resolution, for the same region of chromosome 7 (genomic region: 95.4 to 96.5 Mbp), further validating the approach.

As expected, the ensemble calculations exhibit smoother power-law scaling than their single-cell counterparts. This arises from the large variability among individual chromosome conformations across different samples cells, which is averaged out in the ensemble, yielding a more stable and continuous curve. In contrast, single-cell Hi-C maps are significantly sparser due to limited sampling in any one cell, leading to noisier ligation frequency profiles. This distinction is apparent when comparing the ensemble and single-cell ligation maps, showcasing the effects of averaging over ensembles of different cells with sample-to-sample fluctuations. Nevertheless, Figure 3.(B) provides evidence of power-law-like behavior even in the single-cell case, despite increased noise, supporting the idea that scaling properties are preserved even in sparsely sampled single-cell data.

The noisy curves in Fig. 3(B) already reflect a degree of smoothing, owing to the aggregation of many in-silico scHi-C iterations performed on the same initial native structure. In each iteration, the subsets of nucleosomes selected for crosslinking and bonds chosen for cleavage are independently sampled, introducing statistical variability. Also, the subdiffusive trajectories of polymer segments, driven by Langevin dynamics, differ across iterations. As a result, the ligation events recorded in each iteration sample diverse dynamic outcomes, even though they originate from the same structure. This stochastic variability across iterations effectively smooths the aggregated ligation frequency curve. Figure 3.(F) illustrates the smoothing effect by comparing the average ligation frequency from repeated scHi-C simulations with the underlying contact probability map of the same initial structure. The latter is considerably noisier than the former, underscoring how non-equilibrium dynamics and independent stochastic sampling can average out iteration-to-iteration fluctuations, even when all experiments probe the fixed native conformation.

### Crosslinking chromatin to a nuclear protein network yields maps consistent with experimental results.

Results discussed thus far in Figs. 2 and 3 show that the in-silico protocol, under the assumption of the existence of a nuclear matrix, is consistent with experimental results from actual 3C-based methods. Ligation maps with checkerboard patterns, domains and compartments, mirror those observed in ensemble-average contact and Euclidean maps of the native ensembles; and the dependance of ligation probabilities on genomic distance recovers the expected power-law scaling. While this agreement supports the plausibility of a nuclear matrix as the structural backdrop for crosslinking, it does not exclude alternative mechanisms. In particular, the opposite limiting case—crosslinking via short-range protein bridges—may also yield results consistent with experimental data, and thus remains a viable possibility.

### In-silico Hi-C enables estimation of ligation frequency as a function of Euclidean distance, revealing a typical length scale for ligation events.

While Hi-C experiments typically estimate the ligation frequency as a function of genomic distance, a different but related question remains unanswered: what is the ligation frequency as a function of Euclidean distance? Figs. 3.(C) and 3.(D) address this question with the in-silico protocol. Each data point in these curves represents the probability that two free segment ends -initially separated by a Euclidean distance *“d”* in the native structure- will, at some point in time, come into in physical proximity (within *r* = 1.5*σ* = 15 [nm]) and ligate, given a ligation rate *“p”* (ligation probability per unit time). The resulting ligation frequency curves exhibit a sigmoidal profile, suggesting the existence of a characteristic length scale -on the order of ~20[nm] or so- within which the ligation of free segment ends predominantly occur. This finding is consistent with the proximity-driven nature of 3C methods, where fragment ends already close in 3D space are more likely to ligate than those initially distant. Distant loci must rely on sub-diffusive motion to encounter each other, rendering such ligations statistically less frequent. The estimation of the sigmoid curve, which highlights the local character of ligation events and their typical spatial range, is thus a novel insight accessible through in-silico Hi-C experiments and yet to be probed experimentally.

### Non-equilibrium effects of subdiffusive time evolution manifest through changes in power-law exponents of ligation frequencies versus genomic distance.

**Figure 4.**
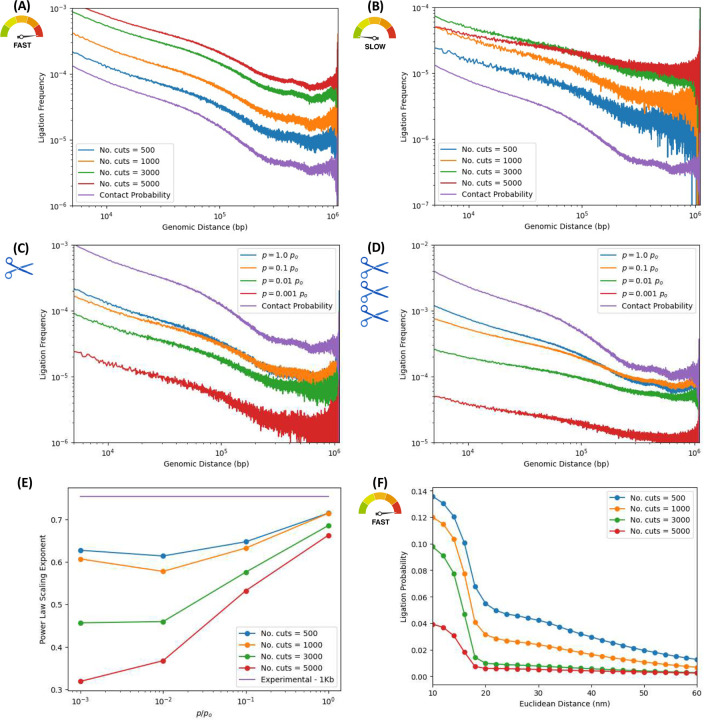
Impact of digestion efficiency on ligation frequencies across different ligation rates. In-silico ensemble Hi-C simulations were performed with 500 crosslinks, varying the number of enzymatically cleaved bonds (“No. cuts”: 500 to 5000) and ligation rates spanning orders of magnitude. Contact probability of native ensemble is shown for refernce. ***(A and B) Ligation frequency vs. genomic distance for varyinf digestion efficiencies:*** We explore non-equilibrium effects of fragment diffusion on ligation maps, in fast and slow ligation rate limits, for different digestion efficiencies. (A) Fast ligation rate (*p* = 1 × *p*_*o*_): Power-law exponents remain largely unaffected by digestion efficiency. Rapid ligation prevents significant fragment diffusion, preserving structural information.. (B) Slow ligation rate (*p* = 10^−3^ × *p*_*o*_): Power-law exponents decrease and curves flatten with increasing digestion. Fragment diffusion erodes structural features in ligation maps. ***(C and D) Ligation frequency vs. genomic distance for different ligation rates:*** Ligation frequencies versus genomic distance are shown for high and low digestion efficiency and different ligation rates. (C) Low digestion efficiency (500 cuts ≈ 9.1% cleaved): Average fragment size ~11 nucleosomes. Exponents are relatively stable across ligation rates, with minimal structural degradation. (D) High digestion efficiency (5000 cuts ≈ 90.9% cleaved): Average fragment size ~1.1 nucleosomes. The polymer approaches a nucleosome gas regime, with diffusive motion erasing structural features—especially at low ligation rates. ***Effect of digestion efficiencies on power-law exponents of ligation frequencies vs. genomic distance:*** (E) Exponents of ligation frequency vs. genomic distance as a function of ligation rate for different digestion levels. Exponents decrease with higher digestion and slower ligation, consistent with (A–D). (F) Sigmoidal decay of ligation frequency versus Euclidean suggests a characteristic distance of ~20 [nm] when *p* = 1 × *p*_*o*_. Ligation frequencies are ordered according to number of cuts, with large digestion efficiency corresponding to lower frequencies. Created in BioRender. Zubillaga, B. (2025) https://BioRender.com/pc4u4ij

To assess how chemical kinetics influence proximity ligation assays such as ensemble Hi-C, we performed in-silico experiments varying two key parameters -digestion efficiency and ligation rate- while keeping the number of crosslinked nucleosomes fixed. Figures 4.(A) and 4.(B) illustrate the resulting power-law scaling of ligation frequency versus genomic distance for the fastest and slowest ligation rates explored in this work, respectively, i.e., *p* = 1 × *p*_*o*_’ and *p* = 10^−3^ × *p*_*o*_.

In each case, plots are shown for different digestion efficiencies, quantified by the number of cleaved bonds per sample (ranging from 500 to 5000), mimicking the activity of restriction enzymes or endonucleases. For “fast” ligation (Fig. 4.(A)), the resulting ligation frequency curves exhibit similar behavior across digestion levels, with consistent scaling exponents close to that of the contact probability for the native ensemble. This reflects the fact that ligation events occur quickly—fragment ends are ligated before significant diffusion can occur, so ligation maps closely reflect the underlying chromatin structure.

In contrast, for slow ligation (Fig. 4.(B)), the scaling exponent becomes increasingly sensitive to digestion efficiency. As more bonds are cleaved, the power-law curves progressively flatten, indicating a decrease in the exponent. This is a consequence of the non-equilibrium nature of the experiment: slower ligation rates grant fragments more time to move away from their initial positions via subdiffusive trajectories before ligating. With increasing digestion efficiency, the number of mobile fragments grows, further amplifying this effect. Thus, in the slow-ligation regime, subdiffusive motion significantly alters the ligation accumulation process, shifting ligation frequencies away from the contact probability of the native ensemble and modifying scaling exponents in a digestion-dependent manner.

This non-equilibrium effect is also shown in Figs 4.(C) and 4.(D), which show the dependance of ligation frequencies on genomic distances for the lowest and highest digestion efficiencies explored in this work, i.e, 500 and 5000 bond cuts, respectively.

Fig. 4.(C), corresponding to the lowest digestion efficiency, shows the ligation frequency scaling laws for several ligation rates spanning orders of magnitude. With only 500 digestions per sample -corresponding to ~9% of linker DNA cleaved- average fragment sizes are some ~11 nucleosomes in length. These relatively long fragments, given the fixed number of 500 crosslinks, help preserve the conformational structure of the initial native ensemble.

Fig. 4.(D), however, corresponds to the highest digestion efficiency, for the same set of ligation rates. Here, 5000 bonds are digested -corresponding to ~90% of the linker DNA cleaved-resulting in average fragment sizes of roughly one nucleosome in length. This produces a “gas” of nucleosomes, which diffuse away from their initial positions with dynamics approaching normal diffusion. The consequence of this irreversible process is the effacing of structure from the Hi-C map, as the spatial organization of the native ensemble is lost to diffusion. Without a corresponding increase in the number of crosslinked beads, higher fragmentation produces smaller fragment lengths, leading to structural degradation in the ligation maps and their associated ligation frequency curves. This progressive erasure of structure pushes the Hi-C map towards a constant matrix with increasing degradation; and the power-law scalings flatten accordingly.

To better assess the non-equilibrium effects of the subdiffusive motion, Fig. 4.(E) quantifies power-law exponent of ligation frequencies versus genomic distance, obtained from least-squares fits to power laws across varying ligation rates (spanning orders of magnitude) and digestion efficiencies (from ~9–90% of linker DNA cleaved), with fixed crosslinking efficiency. Curves corresponding to different digestion efficiencies show the dependance of the exponent on the ligation rate. As before, fast ligation rates enable rapid accumulation of ligation events and an efficient population of the ligation map, which reflects the spatial structure of the native conformations. Slow rates introduce a time delay that allows fragments to undergo significant subdiffusive motion, thereby degrading spatial information of the map and flattening the power-lar scaling. This degradation is exacerbated by increased digestion efficiencies, as smaller average fragment sizes diffuse more freely, eroding the structural information encoded in the original 3D architecture of the native ensemble. The strongest degradation in the power-law exponent is thus observed in the nucleosome gas regime, where digestion is maximal and ligation is slow. Further analysis of these non-equilibrium degradation effects is provided in the Supplementary Information (Figs. S3, S4 and S5).

Numerical estimates of degradation effects in ancient DNA, modeled with diffusive dynamics in nucleosome “gases”, have been used to investigate the surprising preservation of genome architecture in PaleoHi-C experiments on woolly mammoth skin cells.^37^ These diffusion-based models of structural loss are consistent with the degradation trends observed in Fig. 4.

### Increasing crosslinking to nuclear protein matrix helps preserve 3D genome architecture.

**Figure 5.**
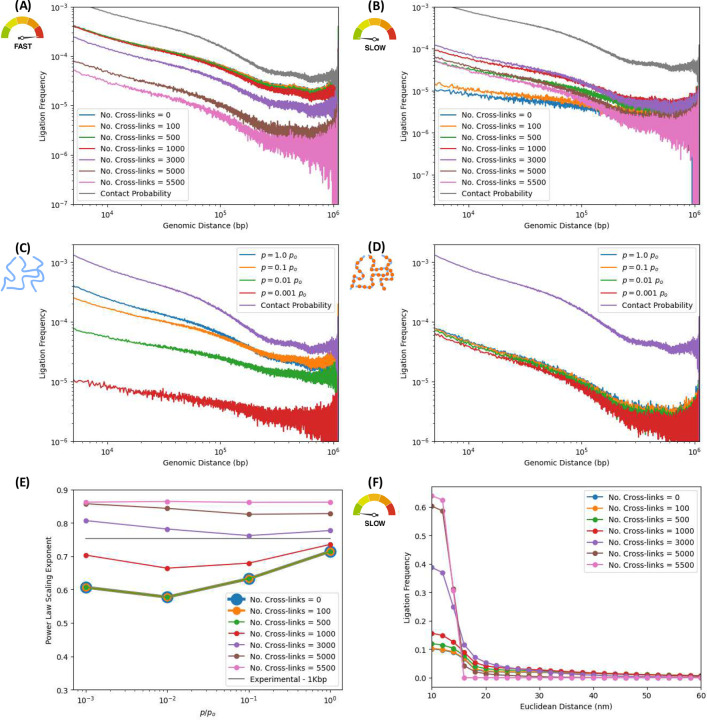
Crosslinking efficiency effects for different ligation rates. In-silico Hi-C ensembles with 1000 digested bonds and varying crosslinking and ligation efficiencies. “No. Cross-links” denotes the number of nucleosomes fixed to the nuclear matrix, from 0 (no crosslinking) to 5500 (fully crosslinked). Ligation rates span several orders of magnitude. Native ensemble contact probability shown for reference. ***(A and B) Ligation frequency vs. genomic distance for different ligation rates:*** We explore fast and slow ligation rate limits, for different crosslinking efficiencies. (A) Fast ligation (*p* = 1 × *p*_*o*_): Power-law exponents remain largely invariant with crosslinking, as ligations occur rapidly before fragments diffuse. (B) Slow ligation (*p* = 10^−3^ × *p*_*o*_): Decreasing crosslinking efficiency leads to progressively flatter scaling curves, due to diffusive displacement of fragments before ligation. ***(C and D) Ligation vs. genomic distance for different crosslinking efficiencies:*** We consider low and high crosslinking efficiencies, for different ligation rates. (C) No crosslinking: Absence of tethered nucleosomes permits diffusion-driven degradation of structure, reflected in flattening of scaling curves at low ligation rates. (D) High crosslinking (~91% of nucleosomes crosslinked): Motion of fragments is arrested; ligation maps retain structural integrity across ligation rates. ***(E) Digestion efficiency effects on power-law exponents of ligation frequencies vs. genomic distance:*** For low crosslinking efficiencies (0–500 crosslinks, i.e. 0–9.1% of nucleosomes crosslinked), exponents decay with lower ligation rates. For high crosslinking, exponents are stable, showing robust structural preservation. ***(F) Effect of crosslinking efficiency on the ligation frequency vs. Euclidean distance:*** Ligation frequency decays sigmoidally with a typical distance for varying crosslinking efficiencies and a low ligation rate *p* = 10^−3^ × *p*_*o*_. Ligation frequencies are ordered according to crosslinking efficiency. At low crosslinking, diffusion allows ligations between more distant fragments. At high crosslinking, motion is suppressed, resulting in sharp decay beyond ~15 [nm]. Created in BioRender. Zubillaga, B. (2025) https://BioRender.com/eir7kx6

Figure 5 illustrates the impact of crosslinker concentration on ligation maps by varying the crosslinking efficiency. Figs. 5.(A) and 5.(B) present ligation frequencies versus genomic distance for a range of crosslinking efficiencies, under fast and slow ligation rate conditions, resp., with fixed digestion efficiency. The crosslinking efficiencies span from zero (no nucleosomes crosslinked) to full immobilization (all nucleosomes crosslinked to the nuclear protein scaffold).

Fig. 5.(A) shows that, in the fast ligation rate limit (*p* = 1 × *p*_*o*_), the power-law exponent remains largely insensitive to crosslinking concentration changes. This reflects the short timescale of ligation events, which occur before significant diffusion, yielding excellent agreement with the contact probability of the native ensemble of structures.

Fig. 5.(B) shows that in the slow ligation rate regime (*p* = 10^−3^ × *p*_*o*_), crosslinking efficiency plays a critical role in preserving chromatin structure. Higher crosslinker concentrations—quantified by the number of nucleosomes tethered to the protein matrix—better arrest fragment motion and maintain the native chromatin conformations, as reflected in power-law scalings that closely match the native ensemble’s contact probability. In contrast, low crosslinking allows free, digested fragments to diffuse away from their original positions, leading to non-equilibrium degradation of structural information and the concomitant flattening of the ligation frequency curves. Thus, reduced crosslinking efficiency progressively deteriorates map fidelity at fixed ligation and digestion conditions.

This effect is further illustrated in Figs. 5(C) and 5(D), which show ligation frequency scaling for varying ligation rates in the extreme cases of no crosslinking and high crosslinking (~91% of nucleosomes tethered), respectively. In the absence of crosslinking, structural degradation becomes evident as a progressive flattening of the power-law curves with decreasing ligation rates, reflecting increased fragment mobility and loss of native spatial information. Conversely, under high crosslinking conditions, fragment motion is effectively arrested as most nucleosomes are tethered to the protein meshwork, yielding ligation frequency scalings that closely match the native contact probability across all ligation rates—demonstrating robust preservation of 3D genome architecture.

Fig. 5.(E) quantifies the effect of crosslinking efficiency on power-law exponents across different ligation rates, with digestion efficiency held constant. At low crosslinker concentrations (<10% of nucleosomes crosslinked), fragment mobility remains largely unconstrained, and exponents decline with decreasing ligation rate, reflecting structural degradation, as fragment motions is not arrested by the nuclear matrix. In contrast, at high crosslinking efficiencies, exponents are well preserved across all ligation rates, as the immobilization of fragments by the protein meshwork maintains the integrity of the native 3D genome architecture.

In the high crosslinking limit, ligation frequency versus Euclidean distance exhibits the characteristic sigmoidal profile (Supplementary Fig. S6), with high frequencies at short distances dropping sharply beyond the typical interaction length scale. Due to the arrested nucleosome motion, spatial organization of the native structures is captured by proximity ligations. The effect of varying the crosslinking efficiency on the sigmoidal decay, in the slow ligation rate limit for a fixed digestion efficiency, is shown in Fig. 5.(F). The arresting effect of high crosslinking concentrations produces sharply decaying sigmoids. As crosslinking decreases, the curves broaden and decay more smoothly, reflecting increased fragment mobility and ligations occurring between loci that have diffused from their native positions.

Further analysis of non-equilibrium effects of fragment diffusion and the motion-arresting role of crosslinks on contact maps, contact probabilities, and power-law scaling is provided in the Supplementary Information (Figs. S7–S9)

### Effect of numerical postprocessing of ligation maps with Knight-Ruiz matrix balancing algorithm is minor relative to raw data.

We assess the impact of post-processing ligation maps using the Knight-Ruiz (KR) matrix balancing algorithm—a widely adopted normalization procedure in 3C methods. KR efficiently enforces double-stochasticity on symmetric matrices, ensuring individual rows and columns of a Hi-C matrix sums to the same value.^38,52,55^ A mean-field assumption of sorts, individual loci are thought to have similar average numbers of contacts. This process is grounded in a mean-field assumption (of sorts) that individual loci exhibit statistically similar average contact frequencies, rendering locus-to-locus variations negligible leading to statistically significant homogeneity across the chromosome. Comparison between raw and KR-balanced maps produced by the in-silico Hi-C protocol reveals minor deviations, indicating that post-processing has a limited effect on the structural information captured by the maps.

**Figure 6.**
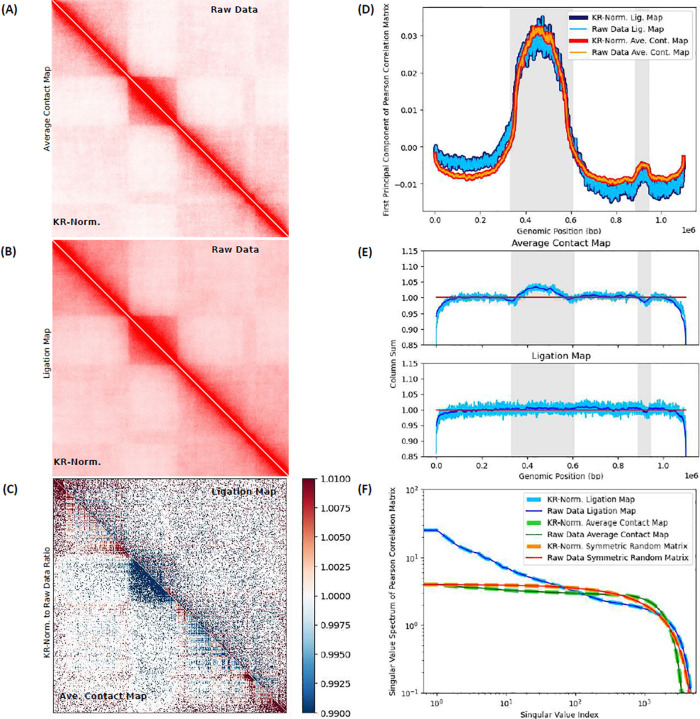
Knight-Ruiz (KR) normalization effects on Hi-C maps. Matrix balancing effects are studied, contrasting KR-normalized (post-processed) with non-normalized (raw) maps. In-silico ligation rate *p* = 10^−1^ × *p*_*o*_, 3000 bond cuts and 500 crosslinks are considered. ***(A) Raw and KR-normalized average contact maps*.** The upper triangular matrix (UTM) displays the raw contact map of the native ensemble. The lower triangular matrix (LTM) shows its KR-normalized counterpart. No visually obvious difference exists between them. ***(B) KR-normalized and raw ligation maps*.** The UTM and LTM display raw and KR-normalized ligation maps, resp. As with (A), no visual difference is apparent. ***(C) Ratio of KR-normalized to raw matrix for ligation and average contact maps*.** The UTM and LTM show the ratio of KR-normalized to non-normalized matrices for ligation and contact maps, resp. Normalization depletes the contact map’s central domain (dark blue), enriching peripheral regions (red). This redistribution is less pronounced in the ligation map, likely due to non-equilibrium diffusion after fragmentation. ***(D) First Principal Component (FPC) of Pearson Correlation Matrix (PCM)*.** The FPC for raw and KR-normalized map overlap for both average contact and ligation maps, indicating that compartment structure is preserved. Gray shading marks compartment switches. (see 6.(A) and 6.(B)). ***(E) Column sums across loci for average contact and ligation maps*.** Column sum per locus is shown versus genomic position for contact and ligation maps (above and below, resp.) Pre and post normalization sums (light blue and red, resp.), and moving averages over the former (dark blue) are shown. Normalization column sums converge to 1. Column sums over contact map distinguish compartment switches (see gray shaded backgrounds). A visible enrichment around the central domain, as well as a small depletion, agree with the FPC in 6.(D). Noisier column sums for ligation map lack obvious enrichments because of non-equilibrium effects. ***(F) Singular value spectra of PCMs*.** Spectra for KR-normalized and non-normalized data, for ligation and contact maps, and a random symmetric matrix with entries drawn from *U*(0,1), show insensitivity to normalization.

Fig. 6 contrasts raw data from non-normalized maps with their KR-normalized counterparts. In Figs. 6.(A) and 6.(B), upper triangular matrices correspond to unnormalized maps, while lower triangles show their KR-normalized versions. Figs. 6.(A) presents average contact maps over the native ensemble, and Fig. 6.(B) shown in-silico Hi-C ligation maps after crosslinking, digestion, subdiffusive dynamics and ligation. Visual inspection of raw versus corresponding KR-balanced maps do not reveal obvious differences, suggesting that perhaps numerical normalization is justified.

Figure 6.(C) presents the element-wise ratio between KR-normalized and raw data maps. The upper triangle corresponds to the in-silico ligation map, while the lower triangle shows the native ensemble’s average contact map. On the average contact map (not subjected to in-silico Hi-C), KR-balancing drains the contact numbers in the central domain and redistributes them towards the chromosome ends. This redistributive effect is less evident in the ligation map due to non-equilibrium effects from digestion and diffusion of free fragments.

In Fig. 6, we apply Principal Component Analysis (PCA) on the Pearson Correlation Matrix (PCM) of both ligation and average contact maps, before and after KR-normalization. Fig. 6.(D) presents the First Principal Component (FPC) of the raw and KR-normalized maps. The FPCs of ligation and average contact maps successfully capture compartment switches (Figs. 6.(A) and 6.(B)). There is no considerable difference between the FPCs of raw and KR-normalized maps: the corresponding curves overlap, both for ligation and average contact maps. These results indicate that KR-normalization does not significantly distort the underlying structural information compressed by the FPCs.

Fig. 6.(E) shows the map’s column sums versus genomic position, both for the ligation and average contact maps. Light blue curves show the magnitude of locus-to-locus fluctuations relative to the moving average (dark blue). The KR algorithm successfully normalizes the matrices and balanced column sums converge to 1 (red). For the average contact map, raw column sums distinguish compartment switches (alternating white and gray shades). The central domain’s visible enrichment and the slight depletion to the right are consistent with the pattern revealed by the FPC in Fig. 6.(D). While KR-balancing also normalizes the ligation map, raw data is noisier due to non-equilibrium effects of diffusive motion: free segments displace from their original positions in the undigested chromosome until ligations take place. Averages contact maps on crosslinked and digested structures, interrogated at different stages of diffusion, reveal non-equilibrium effects of fragment motions on the progressive effacement of enriched column sums in the central domain (see Supplementary Information S10).

Singular Value Decomposition (SVD) of the PCM is performed on both ligation and average contact maps, before and after KR-balancing. Fig. 6(F) presents the corresponding singular value spectra, along with results for a random symmetric matrix with entries drawn from a standard uniform distribution *(0,1). In all cases, the spectra remain virtually unaffected following KR-normalization, suggesting that the structure encoded in the correlation matrices is preserved under balancing.

Insensitivity of FPCs and SVD spectra to KR matrix-balancing and striking visual similarity between pre and post-processed maps, suggest consistency with the mean-field assumption underlying Hi-C map normalization. However, despite the apparent similarity, other metrics -such as ratios of KR-normalized to raw maps and column sums- reveal subtle effects tied to the irreversible nature of the experiment: contact-enriched domains are depleted at the expense of enrichment in other regions of the map, reflecting a global redistribution enforced by balancing.

## 4. DISCUSSION AND CONCLUSIONS

We developed an in-silico protocol simulating proximity ligation assays methods like Hi-C using molecular dynamics and physical modeling, which mimics the effects of DNA crosslinking, digestion and fragmentation of crosslinked DNA with restrictions enzymes or endonucleases, and subsequent ligations between fragment-end pairs. This framework enables systematic control over key parameters affecting the chemical kinetics of Hi-C experiments -including crosslinking efficiency, digestion rate, and ligation kinetics- providing a computational lens to examine the inner workings, assumptions, and gray areas of chromosome conformation capture methods.

Simulations were performed on ensembles of native chromatin conformations, representative of human lymphoblastoid cells. The protocol successfully reproduces hallmark features of Hi-C maps, including checkerboard patterns, compartments, domains, and power-law dependance of ligation frequency on genomic distance. These outputs provide a consistent platform to investigate how experimental conditions impact the fidelity of structural readouts.

In addition to ensemble Hi-C, the protocol also simulates scHi-C methods, with the added luxury of enabling multiple independent repetitions of the single-cell experiment on the same initial structure, aggregating over all iterations. By aggregating over multiple iterations on the same structure, as well as across different conformations in a structural ensemble, it can assess the role of averaging in mitigating noisiness of ligation maps and frequencies. This feature allows for explicit dissection of the roles of experimental stochasticity and biological variability in shaping observed contact maps. Such insights are difficult to obtain from experimental data alone and underscore the potential of physics-based simulations in understanding Hi-C technologies.

Non-equilibrium effects arising from the subdiffusive dynamics of digested, crosslinked chromatin structures manifest clearly in the power-law scaling dependance of ligation frequencies on genomic distance. Fast ligation rates yield near-instantaneous, proximity-driven ligations that preserve native chromatin architecture, even under suboptimal digestion or weak crosslinking conditions. In contrast, slow ligation allows digested fragments to diffuse significantly before ligating, introducing non-equilibrium effects that degrade structural information—especially in the presence of weak crosslinking. High digestion efficiencies further exacerbate this degradation by generating smaller, mobile fragments more prone to displacement prior to ligation. This manifests as a systematic flattening of the power-law curves, deviating from the contact probability scaling of the native ensemble. In contrast, increasing crosslinking efficiency effectively arrests fragment motion, even at slow ligation rates, thereby preserving the original three-dimensional genome architecture. Under these conditions, the power-law exponents of the ligation frequency curves closely resemble those of the native contact probabilities, indicating enhanced fidelity in structural reconstruction facilitated by the stabilizing role of crosslinks to the nuclear matrix.

The protocol also enables investigation into the impact of numerical post-processing algorithms commonly used in Hi-C data analysis, such as matrix-balancing methods like Knight-Ruiz (KR) normalization. Although no appreciable visual differences are observed between raw and KR-balanced maps, normalization redistributes contact counts: depleting enriched regions while enriching others to enforce matrix double-stochasticity. Despite this redistribution, the effects of KR normalization on key structural metrics appear minimal. Similarly, the first principal component (FPC) derived from principal component analysis (PCA) is insensitive to KR balancing, indicating that major compartmental features are preserved. These findings suggest that the raw data satisfies, to a good approximation, the assumption of uniform average contact counts per locus, with only minor locus-to-locus fluctuations. However, compartment-level features can still be detected directly from column sums in the raw contact maps, and measurable shifts in these sums are introduced by normalization. Thus, while PCA-based metrics are robust to KR normalization, other global features -such as count enrichments and depletion- are indeed affected.

In-silico Hi-C enables the investigation of the dependence of ligation probability with respect to Euclidean distance, a relevant aspect of proximity ligation that remains largely unexplored experimentally. Numerical simulations suggest that this dependence follows a sigmoidal trend, characterized by typical ligation length scale in the order ~20[nm]. This underscores the inherently local nature of proximity ligation events and Hi-C maps in capturing the 3D organization of chromatin. As a result of this locality, long-distance and transient interactions are likely underrepresented in ligation-based methods, potentially biasing the inferred genome architecture as revealed by contact maps. In contrast, non-local interactions may be better accounted for by orthogonal and complementary assays such as imaging and GAM (Genome Architecture Mapping), which are not limited by proximity-based ligation.

Numerical experiments demonstrate that modeling DNA crosslinking to an underlying nuclear protein matrix -percolating throughout the nucleus- is consistent with empirical Hi-C data. This concept of such a matrix, entertained in the literature since the 1970s and long surrounded by controversy, has gained renewed interest due to the arrival of recent experimental evidence pointing towards its actual existence.^43,46–51^ While the agreement between our in-silico protocol and experimental Hi-C results do not rule out alternative mechanisms for crosslinking, it lends credence to the hypothesis that DNA may possibly crosslink via long-range bridges through a proteinaceous nuclear scaffold. This meshwork could endow the nucleus with a measure of structural rigidity and contribute to large-scale chromatin organization.

Recently, PaleoHi-C successfully retrieved chromosome conformations from the skin cells of a 52,000 year-old woolly mammoth. A proposed explanation for such remarkable preservation suggests that the nuclear material underwent a phase transition into a glassy state –termed “*chromoglass*”- due to extreme dryness and low temperatures of the Siberian permafrost, effectively fossilizing chromatin architecture.^37^ If protein matrices are indeed genuine structural features of nuclei, perhaps such a matrix contributed to DNA preservation. Specifically, its ability to crosslink DNA oligonucleotides might have helped stabilize chromosome conformations, thereby allowing the three-dimensional genome architecture to survive tens of thousands of years in ancient samples.

Overall, this work suggests that Hi-C maps are not purely equilibrium reflections of spatial proximity, but rather the product of sequences irreversible chemical and physical processes, each of which may be a source of bias. The findings clarify conditions under which structural preservation is favorable, as well as those that may introduce distortions in the interpretation of the results. Thus, our framework serves as a generalizable modeling tool for examining how and to what extent physical and chemical dynamics shape the process of chromosome conformation capture and the final interpretation of its outcomes.

## METHODS

### Ensemble of native structures used in in-silico Hi-C simulations.

An ensemble comprised of 5000 native structures representative of a 1.1Mbp section (genomic region: 95.4 to 96.5 Mbp) of human lymphoblastoid cells at a 200bp resolution was generated with NuChroM, a nucleosome resolution model for Hi-C map inversion trained on experimental ensemble maps.^53^ Code for this model is available at: https://github.com/DiPierroLab/NuChroM.

### In-silico simulator of Hi-C experiments.

The code developed in this project implements an in-silico protocol for proximity ligation assays, effectively modeling crosslinking, restriction enzyme digestion, and ligation processes using the HOOMD-blue molecular dynamics Python library.^56^ In addition, code implementing the FIRE algorithm for preparatory preprocessing, as well as post-processing routines for aggregating ligation events, constructing ligation and contact maps, and computing ligation frequencies as functions of genomic and Euclidean distance, was also developed. All codes is freely available at: https://github.com/DiPierroLab/Bernardo.

### Knight-Ruiz (KR) algorithm for matrix balancing.

A numerical implementation of the Knight-Ruiz algorithm was employed to balance the contact and ligation maps. In particular, the KR code utilized here is provided as a function within the freely available Hi-C Matrix Balancing (HCMB) package.^57^ The original Python implementation of the KR algorithm function contained in HCMB was developed in the context of the gcMapExplorer project.^58^ The KR function from the HCMB repository can be freely accessed at: https://github.com/HUST-DataMan/HCMB.

### Statistical analysis of restriction enzyme digestion sites.

The human genetic sequences analyzed for the study of restriction site statistics were obtained from the Telomere-to-Telomere Consortium. In particular, this work used the T2T-CHM13 reference genome.^59^ The code developed for the analysis of restriction site density and the distribution of distances between consecutive restriction sites in the human genome is freely available at: https://github.com/lrburack/restriction-site-distribution.

### Details on the molecular dynamics of the in-silico protocol for numerical proximity ligation assays.

As was outlined in Section 2 of the main text, the in-silico Hi-C protocol relies on a numerical implementation of crosslinking, digestion, and ligation reactions through molecular dynamics simulations. These processes are modeled within an effective physical framework that the specifies a homopolymer Hamiltonian U_HP_ which depends on the collection of positions $\vec{r}=\left\{{\vec{r}}_{l},\text{i}\in \text{Beads}\right\}$ of all the nucleosome-scale units (beads) in the chromatin polymer. The terms in the Hamiltonian include shifted FENE bonds, truncated Lennard-Jones potentials for hard-core repulsion (volume exclusion) and harmonic angle potentials

$${\text{U}}_{\text{HP}}\left(\vec{\text{r}}\right)=\underset{\text{i}\in \text{Beads}}{\sum }{\text{U}}_{\text{FENE}}\left({\text{r}}_{\text{i},\text{i}+1}\right)+\underset{\text{i},j\in \text{Beads}}{\sum }{\text{U}}_{\text{hc}}\left({\vec{\text{r}}}_{\text{i},\text{j}}\right)+\underset{\text{i}\in \text{Angles}}{\sum }{\text{U}}_{\text{angle}}\left(\theta_{\text{i}}\right)$$

where *Beads* is the set of all beads (nucleosomes), *Angles* is the set of all triplets forming bending angles in the polymer chain, and the aforementioned potentials and bonds are defined as follows:

- Shifted FENE (Finitely Extensible Nonlinear Elastic) bonds *U*_*FENE*_ maintain chain connectivity and prevent unphysical stretching and are defined as:
$${\text{U}}_{\text{FENE}}\left({\text{r}}_{\text{i},\text{i}+1}\right)=\{\begin{array}{ll} -\frac{1}{2}k_{b}\;R_{0}^{2}\;\text{ln}\left[1-{\left(\frac{r_{i,i+1}-R_{\text{shift}}}{R_{0}}\right)}^{2}\right], & R_{\text{shift}}\leq r_{i,i+1}<R_{0}+R_{\text{shift}}\\ 0, & \text{otherwise} \end{array}$$
  with $k_{b}=\frac{30\in }{\sigma^{2}}$, R_0_ = 1.5σ, *R*_shift_ = 1.5σ, ϵ = k_B_T, σ = 1 (10 [nm] in real physical size).
- Hard-core repulsive potentials *U*_*hc*_ between pairs of beads enforce excluded volume effects and are defined by means of truncated Lennard-Jones interactions:
$$U_{\text{hc}}\left(r_{i,i+1}\right)=\{\begin{array}{ll} 4\in \left[{\left(\frac{\sigma }{r_{i,i+1}}\right)}^{12}-{\left(\frac{\sigma }{r_{i,i+1}}\right)}^{6}\right], & r_{i,i+1}<2^{1/6}\sigma \\ 0, & \text{otherwise} \end{array}$$
- Harmonic angle potentials *U*_*angle*_ model bending rigidity and ensure adequate persistence lengths and are defined as:
$$U_{\text{angle}}\left(\theta_{i}\right)=\frac{1}{2}k_{a}{\left(\theta_{i}-\theta_{0}\right)}^{2}$$
  with k_a_ = 4ϵ and *θ*_0_ = *π*.

### Contact and ligation map visualization.

Contact and ligation maps presented in the main body of this work and in Supplementary Information, generated using the in-silico Hi-C protocol, were visualized and inspected with Juicebox, both the web-based as well as the desktop-based visualization apps.^60,61^

## Supplementary Material

Supplementary Files

This is a list of supplementary files associated with this preprint. Click to download.

- SupplementaryInformation.pdf

## Data Availability

The reference genome T2T-CHM13, used for the statistical analysis of restriction enzyme digestion sites is available from Telomere-to-Telomere Consortium, NCBI RefSeq assembly GCF_009914755.1 at https://www.ncbi.nlm.nih.gov/datasets/genome/GCF_009914755.1/. The ensemble of nucleosome resolution structures generated with NuChroM, as well as the molecular dynamics trajectories generated by the in-silico Hi-C protocol, and the results of the statistical analyses including all distance, contact, and ligation maps and ligation and contact probabilities that inform the figures of this paper, are available upon request.

## CODE AVAILIBILITY

The code that implements the in-silico Hi-C protocol, including all preparatory preprocessing and statistical postprocessing steps, is available at https://github.com/DiPierroLab/Bernardo. The code for the analysis of restriction enzyme digestion sites statistics is available at https://github.com/lrburack/restriction-site-distribution. The NuChroM code for generation of nucleosome resolution structures is available at https://github.com/DiPierroLab/NuChroM.
